# An increased incidence of Hodgkin's lymphoma in patients with adult-onset sarcoma

**DOI:** 10.1186/2045-3329-2-1

**Published:** 2012-01-09

**Authors:** Megan E Downing, Gillian S Dite, Mandy L Ballinger

**Affiliations:** 1 School of Medicine, The University of Notre Dame Australia, Sydney, NSW, Australia; 2 Sarcoma Genomics and Genetics Laboratory, Research Division, The Peter MacCallum Cancer Centre, East Melbourne, VIC, Australia; 3 Centre for Molecular Environmental Genetic and Analytic Epidemiology, The University of Melbourne, Carlton, VIC, Australia

**Keywords:** sarcoma, Hodgkin's lymphoma, cancer heritability, second cancer

## Abstract

**Background:**

Sarcomas are rare, often fatal malignancies of connective tissues that can occur in genetic predisposition syndromes or result from carcinogen exposure. Hodgkin's lymphoma (HL) is not known to contribute to any recognised familial cancer syndrome comprising sarcomas, but is known to be associated with a variety of second cancers, including sarcomas. This study describes the prevalence of HL in families affected by sarcoma.

**Methods:**

The International Sarcoma Kindred Study (ISKS) is a prospective cohort of 561 families ascertained via a proband with adult-onset sarcoma. Cancer-specific standardised incidence ratios (SIR) for multiple primary malignancies in probands were estimated. Clinical characteristics of individuals reporting both sarcoma and HL were described. Standardised incidence ratios for the occurrence of cancer in ISKS families were also estimated.

**Results:**

Multiple primary cancers were reported in 16% of probands, significantly higher than in the general population. The risk of HL in probands was increased 15.8-fold (95%CI 7.9-31.6) and increased risks were also seen for breast cancer (SIR 2.9, 95%CI 1.9-4.4) and thyroid cancer (SIR 8.4, 95%CI 4.2-16.8). In 8 probands with both HL and sarcoma, the diagnosis of HL preceded that of sarcoma in 7 cases, and occurred synchronously in one case. Only 3 cases of sarcoma occurred in or close to prior radiotherapy fields. The overall incidence of HL in the ISKS cohort was not significantly increased by comparison with age- and gender-specific population estimates (SIR 1.63, 95%CI 1.05-2.43), suggesting that the association between HL and sarcomas did not extend to other family members. The age of onset of non-sarcoma, non-HL cancers in families affected by both HL and sarcoma was younger than the general population (56.2 y vs 65.6 y, *P *< 0.0001).

**Conclusions:**

The basis for the association between HL and sarcomas may include the carcinogenic effects of therapy combined with excellent survival rates for HL. Common risk factors for both may also exist, including both environmental and heritable factors.

## Background

Cancer is fundamentally a genetic disease. Individual and familial germline characteristics interact with the environment to modify the risk of subsequent somatic genetic changes within cells, the accumulation of which eventually results in cancer development. Cancers often cluster in families, and while some of this clustering may arise from the common environment shared by families, a significant fraction of cancer risk is genetically inherited [1]. Dominant Mendelian hereditary causes are estimated to account for between 1-10% of all cancer, while moderate risk alleles may account for an additional portion of heritable cancer risk [2,3]. In the many pathways contributing to DNA repair systems and cell cycle control, inherited genetic variation and somatic changes to minor susceptibility loci may have a small effect in isolation, but when multiple changes accumulate, may combine to confer a substantially increased cancer risk [1].

Sarcomas are rare, often fatal malignancies of connective tissues whose age of onset is typically younger than most cancers. Sarcomas account for 15% of childhood cancer and 10% of cancer in adolescents and young adults [4]. As a general rule, early age of onset of cancer increases the likelihood genetic factors are present. Sarcomas occur more commonly in a number of recognised hereditary cancer syndromes including Li-Fraumeni syndrome, retinoblastoma, neurofibromatosis type 1, Gardner's and Werner syndromes [5]. Environmental risk factors include exposure to ionising radiation and to a less well understood extent, chemical carcinogens [5,6]. Current knowledge about risk factors for sarcomas is limited. Most studies of heritable risk in sarcomas have focused on paediatric populations, although 90% of sarcomas arise over the age of 15 years [4]. Moreover, the rarity and heterogeneity of sarcomas has presented a barrier to aetiologic study [7].

Another predictor of heritable cancer risk is the presence of multiple primary cancers in individuals [8]. Genetic predisposition and familial aggregation of cancers have been shown to be risk factors for the development of second primary malignancies within individuals [9]. Using a prospectively collected cohort of 561 families affected by adult-onset sarcoma, we identified 87 (16%) probands with multiple primary cancers. In this study we describe the prevalence and types of second cancers in probands with adult-onset sarcoma, and the cancers in their families. An increased risk of second cancers was observed in probands for: HL, breast cancer and thyroid cancer. The characteristics of individuals with a diagnosis of both HL and sarcoma are described in detail.

## Methods

### Design

The International Sarcoma Kindred Study (ISKS) is a prospectively collected familial adult-onset sarcoma cohort, which began recruitment in July 2009. Probands were aged at least 15 years when diagnosed with a histologically confirmed sarcoma or an intermediate aggressive connective tissue tumour and were recruited to the ISKS through sarcoma treatment centres around Australia. The Database contains family history, clinical, epidemiological, pathologic and mutation information. The ISKS Steering Committee granted access to the Database for the purposes of this study under an ethically approved protocol (PMCC HREC Project 09/11).

### Cancer Verification

Wherever possible, reported cancers were verified by hospital or pathology records, physician correspondence or through Australian and New Zealand cancer registries. Validation studies show self-reporting of cancer to be accurate [10,11] and self-report was considered verified in this study. Ninety five per cent of cancers in ISKS probands and 24% of cancers in relatives were verified. In families reporting an incidence of HL, 100% of sarcomas in probands and 49% (75/154) of all cancers were verified. The ISKS attempts to maximise accuracy of reports by recruiting family members of probands. This way, ascertainment of family history is strengthened and multiple first-degree relatives often provide cancer reports. Positive predictive value (PPV) and probability of agreement of reported cancer (with verification source) in first-degree relatives are greater than 75%, with many greater than 90%, for most cancer types including; breast, ovary, prostate, colorectal, pancreas, lung, lymphoma (PPV 85.7%, 95%CI 71.6-99.8) and leukaemia [11]. Of the 69 cancers in the HL families for which documented verification was obtained, 98.5% confirmed self and, or relative report to be correct.

Of the remaining unverified cancers, 11% ISKS and 14% HL families were first-degree reports and 21% ISKS and 17% HL families were second-degree reports. 10% HL family members were overseas and could not be traced. Many reported cases occurred before cancer registries collected data or records had been destroyed. A small number of relatives could not be contacted. Non-melanoma skin cancers and benign tumours were excluded from the analyses. All participants provided written informed consent and ethics approval was obtained from all relevant research ethics committees.

For cancers without verification and where age data was unknown, the following protocol was used to obtain estimates: Date of birth; estimated 25 years before the date of birth of the oldest child. If an individual had no children, then date of birth estimated 25 years after their mother's date of birth. Age at death; estimated 70 years, or if born after 1951 date of death estimated as 2010. For age at cancer diagnosis; if an individual was alive and born before 1951, estimated as 60 years. If deceased, date of diagnosis estimated four years before their date of death. These rules were applied to estimate ages in 348 cancers within the ISKS cohort.

### Statistical methods

Australian population-based cancer incidence rates specific for cancer site, age and year of birth (both in five-year groupings) were obtained for 1982 to 2007 from the Australian Institute of Health and Welfare [12]. In ISKS probands, the standardised incidence ratio (SIR) for each cancer site (excluding sarcomas) was estimated by comparing the observed number of cancers to the number expected from population incidence rates. We then sought to assess the cancer profile in ISKS families compared to the general population in the same method. We age-adjusted the Australian population-based incidence data to correct for the selection bias inherent in recruiting families for the incidence of sarcoma, a malignancy of young age of onset. Mean difference in age at onset was calculated using a one-sample t-test against Australian population data. 95% confidence intervals were calculated for all results. Statistical analyses were performed with Stata version 11 (StataCorp, 2011). All statistical tests were two-sided and *P *values < 0.05 were considered nominally statistically significant.

## Results

**Figure 1:**
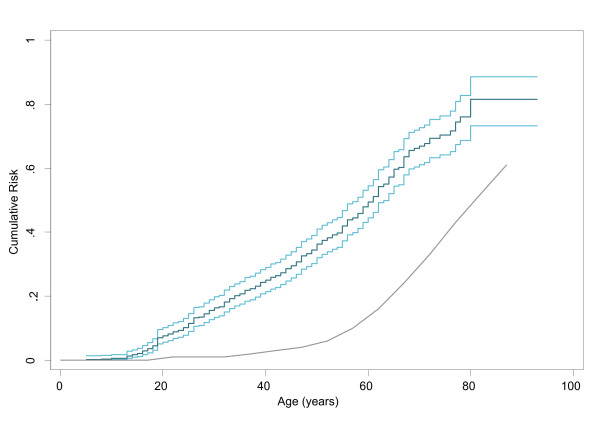
**Cumulative risk for all cancers in ISKS probands (excluding sarcoma)**. Green = ISKS probands curve Light Blue = 95% confidence interval Grey = AIHW population curve.

Overall, 2097 cancers were reported in 561 ISKS families, and included 561 sarcomas in probands and 1536 other cancers. Multiple primary malignancies were seen in 87 (16%) probands (see Table 1). In probands, there was an increased risk of HL (SIR 15.8, 95%CI 7.9-31.6), breast cancer (SIR 2.9, 95%CI 1.9-4.4) and thyroid cancer (SIR 8.4, 95%CI 4.2-16.8). There was an increased risk of cancer other than sarcoma in ISKS probands and the cumulative risk is shown in Figure 1, reinforcing the impression that sarcoma is associated with an increased incidence of other primary malignancies.

**Table 1:** Primary malignancies in ISKS probands (excluding sarcoma)

Cancer	O	E*	SIR	95% CI
Hodgkin's Lymphoma	8	0.51	15.8	7.9-31.6
Thyroid	8	0.95	8.4	4.2-16.8
Breast	21	7.3	2.9	1.9-4.4
Lung	10	4.9	2.1	1.1-3.8
Colorectal	8	6.7	1.2	0.6-2.4
Prostate	6	8.11	0.7	0.3-1.6

*AIHW 1982-2007

CI confidence interval, E expected, O observed, SIR standardised incidence ratio

Given the increased risk of HL in probands with sarcoma, we focused on families affected by HL in more detail. Twenty two families (4%) reported at least one case of HL. Of the 22 cases of HL in these families, 8 occurred in probands (6 male, 2 female), and 14 in relatives (4 male, 10 female). To determine whether the overall incidence of HL and other cancers in the ISKS cohort was increased, the cancer profile in all 561 ISKS families was compared to an age- and gender-adjusted Australian population. An increased standardised incidence ratio for brain (SIR 1.8, 95%CI 1.37-2.33, *P *< 0.001) and breast (SIR 1.25, 95% CI 1.10-1.41, *P *< 0.001) cancers was observed. A small, but non-significant increase was indicated for HL (SIR 1.63, 95%CI 1.05-2.43). These data indicate that the increased incidence of HL in probands was not reflected in an increased incidence of HL in family members.

In families affected by both HL and sarcoma, 110 non-sarcoma, non-HL cancers were reported in 88 people. The mean age (± standard deviation) at diagnosis of all 110 cancers was 56.2 ± 17.7 years, significantly younger than the general population (mean 65.6 ± 15.2 years; *P *< 0.0001)[13]. Among the 110 cancers, 21 (19%) occurred before the age of 45 years, compared to 8.6% of all cancers occurring before 45 years in the Australian population. Eighteen individuals reported two primary cancers (eight probands, ten relatives) and two reported three primary cancers (two probands).

The increased incidence of HL in probands suggests either a common predisposition to both sarcoma and HL or carcinogenic effects of therapy for previous cancer diagnosis. To assess potential effect of prior cancer therapy in the development of sarcomas following HL, detailed clinical information for the 8 probands affected by both HL and sarcoma was collected (Table 2). In all but one case, the HL was diagnosed prior to the sarcoma, with a median latency of 19 years (range 9 - 32 years). One case had synchronous diagnoses of HL and sarcoma. Only three of the eight sarcomas occurred within or adjacent to the HL radiotherapy field, while another three sarcomas arose at sites distant to the radiation field, and one case was treated with chemotherapy only.

**Table 2:** Hodgkin's lymphoma and sarcoma in 8 probands

Case	Gender	HL age onset	HL subtype	HL site	HL treatment		Other primary malignancy	Sarcoma age onset	HL - Sarcoma Latency (years)	Sarcoma subtype	Sarcoma site	Relation to HL RT
					CT	RT						
1	M	52	Nodular sclerosing	Neck	ABVD 4 cycles	MFRT 30.6 Gy 17#	Melanoma L UL 59 y excision	61	9	Fibromyxosarcoma	R thigh	Not in field
2	M	44	Nodular sclerosing	Head/neck	NM 6 cycles	35 Gy 20#	-	69	25	Fibromyxosarcoma	R UL	In field
3*	M	34	Mixed cellularity	R axilla	MOPP + ABVD 8 cycles combination	MRFT 38 Gy 27#	Melanoma UL 47 y excision	60	26	Pleomorphic cell sarcoma NOS	L latissimus dorsi	In field
4	M	51	Nodular sclerosing	Chest	ABVD	IFRT dose unknown	-	62	11	Leiomyosarcoma	Pelvis	Not in field
5	F	33	Unknown	L axilla	CT treatment records unavailable	RT dose unknown	-	52	19	Osteosarcoma	L latissimus dorsi	In field
6	M	19	Nodular sclerosing	Chest, lungs	ABVD 1 cycle	-	-	19	0	Chondroblastic osteosarcoma	R LL	No RT Synchronous diagnoses
7	F	18	Nodular sclerosing	Chest, abdomen, pelvis	CT	-	-	27	9	Clear cell sarcoma	Small bowel	No RT
8	M	22	Mixed cellularity	Neck, chest, abdomen	CT	36 Gy 18#	-	54	32	Malignant fibrous histiocytoma	L thigh	Not in field

* *TP53 *mutation positive

ABVD Adriamycin, bleomycin, vinblastine, dacarbazine, CT chemotherapy, F female, Gy gray, IFRT Involved field radiotherapy, HL Hodgkin's lymphoma, L left, LL lower limb, M male, MFRT mantle field radiotherapy, MOPP Mustargen, Oncovin, Procarbazine, Prednisone, NM Nitrogen mustard, NOS not otherwise specified, RT radiotherapy, R right, UL upper limb

Histological subtypes of HL reported in probands were nodular sclerosing (5/8), mixed cellularity (2/8) and one unknown. This is consistent with expected rates of the spectrum of histological subtypes [14]. One proband with HL reported a synchronous diagnosis of osteosarcoma. A variety of subsequent sarcoma subtypes were reported by seven probands with HL and included: two fibromyxosarcomas, one leiomyosarcoma, one clear cell sarcoma of the small bowel, one osteosarcoma, one malignant fibrous histiocytoma and one pleomorphic cell sarcoma not otherwise specified. Two probands who reported both HL and sarcoma also reported malignant melanomas, without nodal involvement and for which neither required radiotherapy or chemotherapy. The remaining 14 sarcoma probands in the HL families also reported a variety of histological subtypes, which included: leiomyosarcoma (4/14), Ewing's sarcoma (2/14), and one each of osteosarcoma, fibrosarcoma, clear cell chondrosarcoma, epithelioid haemangioendothelioma, haemangiosarcoma and synovial sarcoma as well as two soft tissue sarcomas not otherwise specified. Two of these 14 probands also reported other cancers. One reported carcinoma-in-situ of the breast, for which she had radiotherapy. A lower limb sarcoma developed 10 years later, outside the radiotherapy field. One other proband reported an earlier diagnosis of acute lymphoblastic leukaemia (latency 6 years), for which she was treated with Berlin-Frankfurt-Münster 1995 protocol chemotherapy, without irradiation. This protocol is associated with a cumulative risk for second malignant neoplasms of 3.3% after 15 years, but only 1.2% in non-irradiated patients [15]. Acute myeloid leukaemias and central nervous system tumours accounted for 70% of second cancers in this study.

The ISKS cohort is being systematically screened for mutations in the *TP53 *gene (manuscript in preparation) and 3 of the 16 probands in the HL families (including one who had both a sarcoma and HL) were positive for germline mutations in *TP53 *(19%).

## Discussion

After familial clustering, second primary malignancies in individuals are a hallmark of cancer predisposition syndromes [16]. The findings presented here are consistent with previous reports of an increased rate of sarcomas in survivors of HL [17,18]. In addition, an increased risk of breast cancers and thyroid cancers was seen in patients with sarcoma. The risk of sarcoma development subsequent to HL has almost exclusively been attributed to the effects of radiotherapy, which is known to cause malignancy, but the absolute risk is low [18-23]. In our cases, five of the eight cases of sarcoma were not attributable to radiation exposure. Increased radiotherapy dose increases risk for sarcoma development (RR 26.6, 95%CI 13.3-47.6)[20], though combination chemotherapy with low-dose radiotherapy is also associated with increased risk (SIR 88.9, 95%CI 24.2-227.6) [24]. A recent publication also showed an increased risk for sarcoma due to chemotherapy plus radiotherapy (SIR 8.9, 95%CI 2.9-20.7), however found no increased risk for sarcoma development due to chemotherapy treatment for HL alone [22]. Our study suggests that radiation exposure does not fully explain the development of sarcoma, which may instead be caused by chemotherapy, a common environmental carcinogen or a common heritable predisposition.

Shared risk factors for the development of lymphoma and sarcoma, including acquired immunodeficiency and herbicide exposure may exist [7]. In a case-control study assessing risk factors for the development of soft tissue sarcoma, excess lymphoma and specifically Hodgkin's (OR 8.9, 95% CI 1.7-51.2) was noted among sarcoma relatives [25]. Our data do not support an increased risk for HL in the ISKS cohort as a whole. However, we have not excluded the possibility of a common predisposition, whether hereditary or environmental, to both HL and sarcoma. Ascertainment biases also underestimate the true rate of cancers in relatives of probands in family cancer studies [26]. The ISKS partially addresses this issue by recruiting first-degree relatives who provide self-reports of cancers. In addition, cancers were verified through access to population registries and by obtaining pathology reports. While our data do not support common predisposition to HL and sarcoma, the one case of synchronous HL and sarcoma cannot be explained by the carcinogenic effects of prior treatment of HL, whether radiotherapy or chemotherapy.

The pattern of second primary cancers in sarcoma-affected probands is of interest. Breast cancer is the most commonly reported primary cancer associated with development of subsequent radiation-associated soft tissue sarcoma [27,28]. The median latency period between radiation exposure and sarcoma development has been reported between 8.4 and 10 years [27,28]. Given breast cancer incidence in the population is significantly higher than HL, we expected a greater proportion of ISKS probands reporting previous breast cancer. We considered an ascertainment bias for survival of the primary cancer, but the overall survival of patients with breast cancer is comparable with HL [29]. The incidence of breast cancer and brain cancer in families with sarcomas is reminiscent of the Li-Fraumeni Syndrome. As in our cohort, the incidence of lymphoma in a US-based case control study of 24 families with Li-Fraumeni Syndrome was not increased [30]. None of the cancer pedigree patterns in the 22 HL families in the ISKS fit the classic criteria for Li-Fraumeni Syndrome [30], or any other familial cancer syndrome. However, the rate of *TP53 *mutations in HL probands (19%) was higher than that reported in the only previous study of *TP53 *mutations in sarcoma-affected populations [8].

## Conclusions

We have presented findings that suggest an association between the development of sarcomas and HL. The increase in risk for HL occurring in sarcoma probands was not adequately explained by prior therapies, and did not extend to relatives.

## Abbreviations

CI: confidence interval; HL: Hodgkin's lymphoma; ISKS: International Sarcoma Kindred Study; OR: odds ratio; PPV: Positive predictive value; SIR: standardised incidence ratio.

## Competing interests

The authors declare that they have no competing interests.

## Authors' contributions

MED participated in the design of the study, data acquisition, analysis and interpretation, and drafted the manuscript. MLB coordinated the study and participated in data analysis and interpretation and manuscript revision. GSD participated in data analysis and interpretation. ISKS contributed data and site investigators conceived of the study and its design and assisted in revision of the manuscript. All authors read and approved the final manuscript.

## Authors' information

The Australian site investigators for the International Sarcoma Kindred Study are Sandro Porceddu, Michael Gattas, Ian Dickinson, Susan Neuhaus, Graeme Suthers, David Thomas, Gillian Mitchell, Martin Tattersall, Craig Lewis, Kathy Tucker and Richard Carey-Smith.
